# Influence of Age and Breed on Bovine Ovarian Capillary Blood Supply, Ovarian Mitochondria and Telomere Length

**DOI:** 10.3390/cells10102661

**Published:** 2021-10-05

**Authors:** Paweł Kordowitzki, Roswitha Merle, Pascal-Kolja Hass, Johanna Plendl, Juliane Rieger, Sabine Kaessmeyer

**Affiliations:** 1 Department of Immunology and Pathology of Reproduction, Institute of Animal Reproduction and Food Research of Polish Academy of Sciences, 10-748 Olsztyn, Poland; p.kordowitzki@pan.olsztyn.pl; 2 Department of Basic and Preclinical Sciences, Institute for Veterinary Medicine, Nicolaus Copernicus University, 87-100 Torun, Poland; 3 Department of Veterinary Medicine, Institute for Veterinary Epidemiology and Biostatistics, Freie Universität Berlin, 14195 Berlin, Germany; Roswitha.Merle@fu-berlin.de; 4 Department of Veterinary Medicine, Institute of Veterinary Anatomy, Freie Universität Berlin, 14195 Berlin, Germany; pascal-kolja.hass@fu-berlin.de (P.-K.H.); Johanna.Plendl@fu-berlin.de (J.P.); juliane.rieger@vetsuisse.unibe.ch (J.R.); 5 Division of Veterinary Anatomy, Vetsuisse Faculty, University of Bern, 3012 Bern, Switzerland

**Keywords:** aging, cattle, ovary, vascularization, capillary, telomere, high producing cattle, dual purpose breed

## Abstract

Worldwide, dairy cows of the type of high-producing cattle (HPC) suffer from health and fertility problems at a young age and therefore lose productivity after an average of only three lactations. It is still contentious whether these problems are primarily due to genetics, management, feeding or other factors. Vascularization plays a fundamental role in the cyclic processes of reproductive organs, as well as in the regeneration of tissues. In a previous study, HPC were shown to have a greater ovarian corpus luteum vascularization compared to dual-purpose breeds. We hypothesize that this activated angiogenesis could likely lead to an early exhaustion of HPC′s regenerative capacity and thus to premature reproductive senescence. The objective of this study was to investigate if a HPC breed (Holstein-Friesian, HF) exhibits higher ovarian angiogenesis than a dual-purpose breed (Polish Red cow, PR) and if this is related to early ovarian aging and finally reproductive failure. For this purpose, we assessed the degree of vascularization by means of ovarian blood vessel characterization using light microscopy. As indicators for aging, we measured ovarian mitochondrial size and telomere length in peripheral leukocytes. We report in this study that in both breeds the distance between capillaries became smaller with increasing age and that the mean telomere length decreased with increasing age. The only difference between the two breeds was that PR developed larger capillaries than HF. Neither a relationship between telomere length, nor the morphology of the mitochondrial apparatus and nor angiogenesis in HF was proven. Although the data trends indicated that the proportion of shortened telomeres in HF was higher than in the PR, no significant difference between the two breeds was detected.

## 1. Introduction

The development of various phenotypic and genetic analytical approaches within the past decades has extensively contributed to our current understanding of cattle reproduction. The gained knowledge has provided strong evidence that certain breeding goals, which for an extended period of time were focused on enhanced milk yield, may have negative impact on fertility or susceptibility to diseases [1,2]. Globally, the high-producing cattle (HPC) breed is the leading milk-producing breed [3]. However, it has been hypothesized that high-producing dairy cows, with a severe negative energy balance in the early post-partum period, suffer more often from health and fertility problems at a younger age than other breeds or domesticated animals [4,5]. It is still contentious whether this is mainly due to genetics, management, feeding or other factors. Health and fertility issues in HPC, which are known to be typical signs of aging in other breeds and species (e.g., reduced wound healing or infertility), often result in the early culling of affected cows after less than three lactation periods in average [6]. Decreased fertility and pre-term culling results in high economic losses to the dairy industry and has a negative impact on sustainability and climate protection [7]. An intact high performing reproductive tract is the key element of a successful dairy breed and so is the circulatory system of these organs. Vascularization plays a fundamental role in the cyclic processes of both the ovary and uterus in all phases of lactation: during the regeneration and remodeling of tissues after calving, during the development of the placenta in pregnant cows and during the dry period [8,9,10]. In the ovary, adequate vascularization is crucial for follicular growth and maturation and therefore, for oocyte and embryo development. Blood vessel formation is likewise prerequisite for the development and sustenance of the mammary gland synthetic capacity. In a previous study, HPC exhibited a greater ovarian vascularization in the corpus luteum compared to dual-purpose breeds [11]. This longtime activated angiogenesis could likely lead to an early exhaustion of HPC′s regenerative capacity, eventually leading to decreased vascularization and thus to premature senescence, after a short lifespan characterized by constant, maximal performance that takes its toll on the body. Indeed, in precocious aging (klotho) mice, a model for early aging, it was found that blood vessel density was initially similar to control mice but blood vessel development was reduced. After the induction of hind leg ischemia, the muscle capillary density was reduced, and impaired angiogenesis was observed in an aortic ring culture test [12]. Apart from angiogenesis, the ultrastructure of the mitochondrial apparatus is another parameter for aging. Adequate mitochondrial dynamics are important for normal cell function since the mitochondrion is the powerhouse of the cell. Disturbed mitochondrial dynamics can therefore influence cell differentiation, proliferation, cell reprogramming and aging. The shape of the mitochondria is closely linked to their function [13]. They form interconnected networks, the plasticity of which is important in order to react to stress and changes in nutrient availability [14]. For the neural mitochondria of *Caenorhabditis elegans*, it has been shown that size, density and resistance to oxidative stress increase in early adulthood, remain at a high maintenance level in mid-adulthood and decrease later in life [15]. The aging of cells can also be assessed by the length of telomeres, the end fragments of (human and animal) chromosomes, which shorten with each cell division (until senescence). Mammalian telomeres consist of repetitive G-rich sequences (TTAGGG) and associated proteins (shelterins), which cap the ends of linear chromosomes and maintain chromosomal stability [16]. In most human somatic cell types, telomeres shorten with each cell cycle due to the absence of telomerase and the end replication problem [17]. 

The objective of this study was to investigate whether there is a relationship between telomere length, the morphology of the mitochondrial apparatus and angiogenesis in HPC and whether there is a difference between HPC (Holstein-Friesians, HF) and a robust dual-purpose breed (Polish Red cow, PR). The overall aim was to find out whether HPC require higher angiogenesis for high performance, but age faster as a result of it. Our study could serve as model for the aging phenomenon in general, since high milk yield reflects a tremendous stressor for the cow, which might lead to an accelerated aging process. 

## 2. Materials and Methods

### 2.1. Animals

In the present study, ten healthy, cycling, Polish Holstein-Friesian cows, (HF, n = 5, reflecting a high-milk-yield breed, age range 62–116 months, median 85) and Polish Red cows (PR, n = 5, reflecting dual-purpose (milk/meat) breed, age range 39–108 months, median 61) were selected for ovarian and blood sampling. Sample types were collected at a local abattoir from a group of healthy, non-pregnant cows (age range 39–116 months) which were considered for culling due to the replacement by heifers according to the breeding and selection program or due to locomotion problems. All cows underwent a general clinical examination and were confirmed to be free of reproductive tract disorders. All procedures related to sample collection were approved as stated in the “Institutional Review Board Statement”.

### 2.2. Blood Collection and Further Processing

Blood samples were obtained via intracardiac aspiration from all donors immediately after culling at a local abattoir. The blood samples were processed with erythrocyte lysis buffer (Qiagen Polska Sp. z o.o., Wroclaw, Poland) according to the manufacturer’s protocol since the telomere measurements were acquired in leukocytes. The samples were frozen at −80 °C slowly in a Nalgene Cryo Freezing Container (Sigma-Aldrich Sp. z o.o., Poznan, Poland).

### 2.3. Ovary Sample Collection 

Ovarian tissue was sampled immediately post mortem at a local abattoir. The ovaries were cut in two halves, and tissue samples (1 cm in length and 0.5 cm in width) of the zona parenchymatosa and zona vasculosa were transferred into transport tubes containing either 4% neutral buffered formalin for light microscopy or Karnovsky′s fixative (7.5% glutaraldehyde and 3% paraformaldehyde in phosphate buffered saline) for electron microscopy.

### 2.4. Sample Preparation for Light and Transmission Electron Microscopy

The specimens for light microscopy were dehydrated in a series of ascending concentrations of ethanol solutions and processed for embedding in paraffin wax. Five μm thick sections were cut and dewaxed using xylene, rehydrated through descending concentrations of ethanol and stained with gallocyanin-, chromotrope 2R- and aniline blue stain (GRA)—a modified trichrome stain for a general overview of tissue morphology and to identify regions of interest in the zona parenchymatosa for lectin-histochemical analysis. Lectin histochemistry was used to label blood vessels in paraffin sections of ovarian samples with Bandeiraea simplicifolia agglutinin I (BSL) according to a previously published protocol [11].

For transmission electron microscopy, samples were processed according to a previously published protocol [18]. In short, semi-thin sections (0.5 µm) were stained with modified Richardson′s solution and then analyzed by light microscopy to identify regions of interest in the zona parenchymatosa. Ultrathin sections of the identified regions were prepared for analyzation via transmission electron microscopy (TEM). 

### 2.5. Capillary Measurement

The sections marked with lectins were scanned with a light microscope (Eclipse Ni-E, Nikon, Düsseldorf, Deutschland) equipped with a color camera (DS-Fi2). The software NIS-Elements AR 5.02 was used for evaluation and measurements. Vascularization parameters were assessed in two areas, the theca interna folliculi of tertiary follicles and in sections of the zona parenchymatosa without recognizable functional structures. In order to clearly identify the zona parenchymatosa and functional structures, HE- and GRA-stained serial sections were used in parallel. The following parameters were measured morphometrically: number of capillaries per area, intercapillary distance, capillary size (diameter), area of the individual capillary lumen and the percentage of the area occupied by capillaries. In the theca folliculi, the whole thecal area was measured. In the zona parenchymatosa without visible functional structures, four areas each with a dimension of 500 × 500 µm were measured. Regions of interest (ROI) were set, in which the capillaries were detected automatically via a color-, size- and form-threshold. The intercapillary distance was measured manually.

### 2.6. Mitochondria Measurement

The size of mitochondria was measured in randomly selected cells of the ovary via TEM using a JEM-1400 Flash electron microscope (JEOL GmbH, Freising, Germany). The following parameters were recorded: the average of +50 measured mitochondrial lengths, which were always the longest uninterrupted measurement line through the mitochondria in nm; the average of +50 measured mitochondrial diameters, which were always orthogonal to the length in nm. The area of the mitochondria in nm^2^ was determined from these values, assuming an eliptic shape. The following formula was used for the measurement: A = a⋅b⋅π −> a,b semi-axes of the ellipse.

### 2.7. High-Throughput Q-FISH for Telomere Length Measurement

Telomere length measurement in peripheral *blood mononuclear cells* was performed as previously described [19]. Briefly, blood samples were thawed quickly and re-suspended in complete RPMI media and plated in poly-L-lysine pre-coated clear bottom black-walled 96-well plates (Greiner, Kremsmünster, Upper Austria, Austria). Samples were analyzed in duplicates. To convert telomeres fluorescence values into kb, we used standard cell lines with stable telomere length: L5178Y-R (79.7 kb), HeLa1211 (21.11 kb), Jurkat (11.5 kb), S (10.3 kb), K562 (6.5 kb), and HeLa R (6.03 kb). Images were acquired on an Opera High Content Screening System (PerkinElmer, Inc., Waltham, Massachusetts, USA) and analyzed with Acapella Image analysis software (PerkinElmer, Inc.).

### 2.8. Statistics

Data were stored in MS Excel Version 2016 and analyzed using IBM SPSS Statistics 25. Correlations between numeric variables were calculated following the Kendall-tau method for nonparametric values and small sample sizes. Multivariable general linear models were used to analyze the influence of age and breed on the vascular and mitochondrial, as well as telomere, parameters. If necessary, the influence parameter was transformed to quadratic terms to achieve linearity. This was the case for the following models:

- Influence of the size (area) of the tertiary follicle on the number of capillaries per area
- Influence of age on the average distance between two capillaries in the tertiary follicle, as well as on the average area per capillary, the average diameter per capillary and on the proportion of the lumen area of the capillaries in relation to the total measuring area in areas without functional structures

Model diagnostics included the visual inspection of linearity and the homoscedasticity of residuals, as well as adjusted R-squared values. *p*-values < 0.05 were regarded significant. 

## 3. Results

### 3.1. Vascularization of the Tertiary Follicles Was Strongly Dependent on the Follicle Size

The follicle sizes (area of the theca interna folliculi) of available tertiary follicles were heterogeneous; they ranged from 0.1 mm^2^ to 1.68 mm^2^ (mean 0.48 mm^2^; median 0.31 mm^2^; SD 0.47 mm^2^). The vascularization of the theca interna of the tertiary follicles was strongly dependent on the follicle size and increased with the increasing size of the follicle in both examined breeds. A positive correlation was found between the area of the theca interna folliculi and the capillary size (individual lumen area: 0.556; *p* = 0.037 and diameter: 0.667; *p* = 0.012), as well as the proportion of the lumen area of all capillaries in the total measurement area (0.333; *p* = 0.211). The measured values for the vascularization of theca interna folliculi for individual cows are shown in Table 1. The statistical models did not show any informative value for age and breed for this measurement area (low adjusted R^2^ values).

### 3.2. Vascularization of the Zona Parenchymatosa without Functional Structures: PR Had Larger Capillaries Than HF and the Distance between Blood Capillaries Became Smaller with Increasing Age in Both Breeds

**Figure 1:**
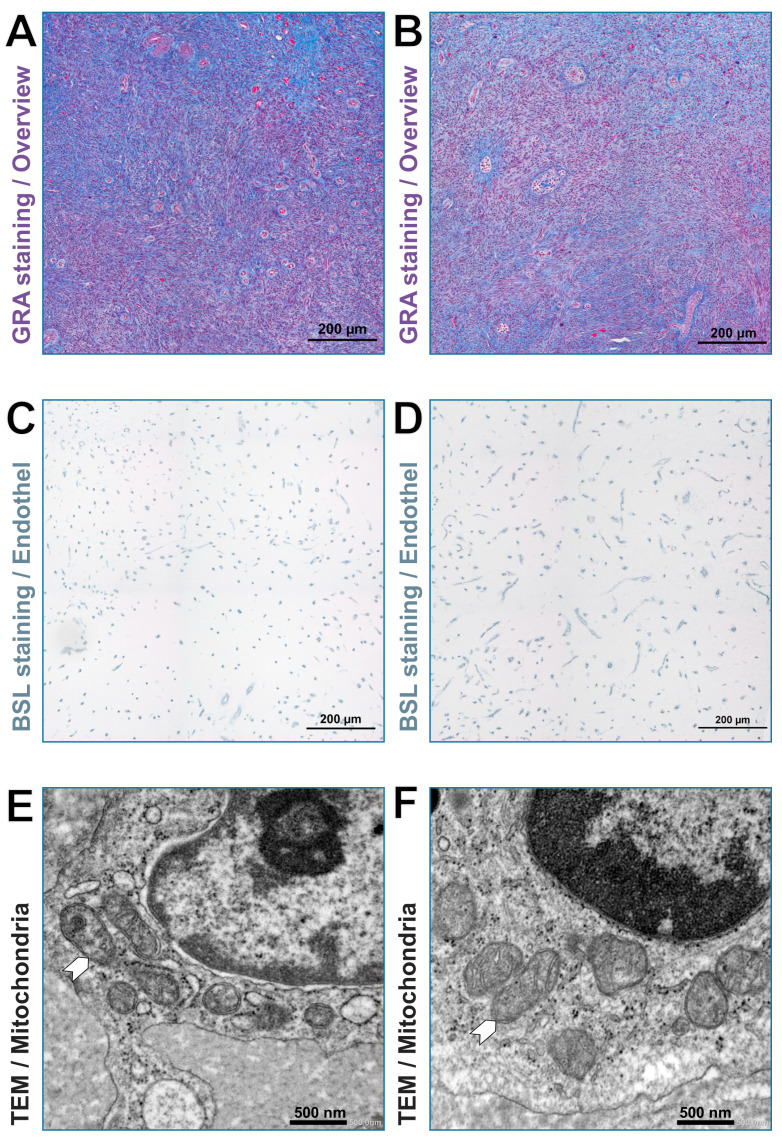
Examples of the measured areas in the ovarian zona parenchymatosa without visible functional structures. **A**,**C**,**E** Holstein-Friesian. **B**,**D**,**F** Polish Red cow. **A**,**B** GRA staining. **C**,**D** BSL staining-endothelial cells marked in dark brown-black. **E**,**F**, transmission electron microscopic picture showing mitochondria (white arrow heads). *Abbreviations*: BSL: Bandeiraea simplicifolia agglutinin I; GRA: gallocyanin-, chromotrope 2R- and aniline blue stain; TEM: transmission electron microscopy.

**Figure 2:**
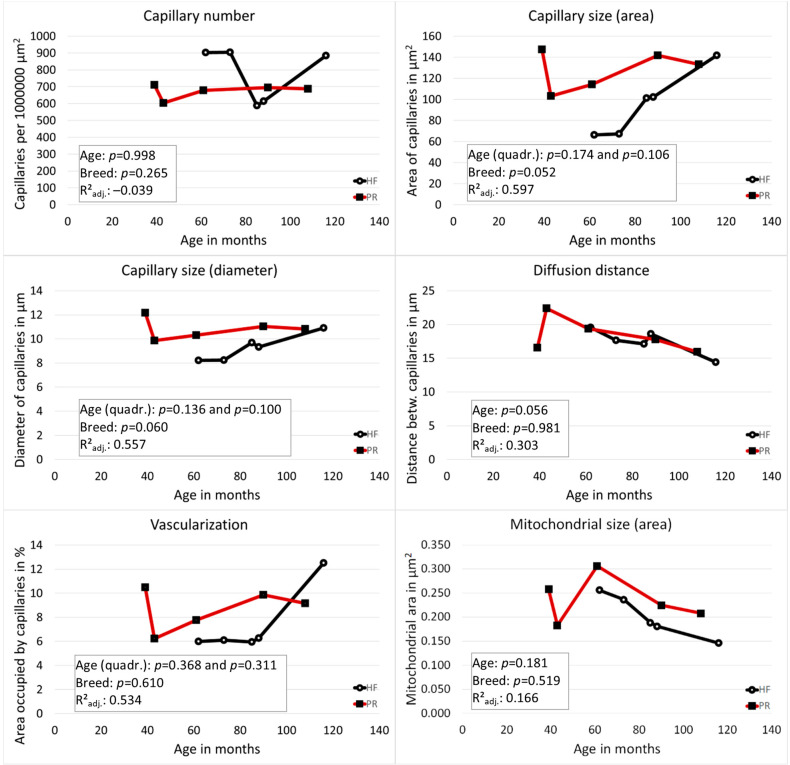
Vascular and mitochondrial parameters measured in the ovarian zona parenchymatosa without visible functional structures. HF (black lines) Holstein-Friesian. PR (red lines) Polish Red cow. Inserted boxes show statistical results from the multivariable general linear model, which was used to analyze the influence of age and breed.

PR had larger capillaries than HF (area *p* = 0.052, diameter *p* = 0.060). The distance between the capillaries became smaller with increasing age in both breeds (*p* = 0.056). A linear influence of age on vascularization (area occupied by capillaries in %) could be shown for both breeds in a linear regression analysis (*p* = 0.027). However, the capillary area initially decreased and then increased again from the age of 70 months (age range of all animals: 39–116 months). In the multivariable general linear model, this quadratic relationship of age and the vascularization turned out to be not significant (*p* = 0.368 for linear, *p* = 0.311 for quadratic term). Representative images from the ovarian zona parenchymatosa without functional structures of the two breeds are displayed in Figure 1. The results referring to the vascularization for the individual cows are shown in Figure 2 and Table 2.

### 3.3. No Correlation between the Vascularization Measurements of the Two Areas

A comparison between the vascularization of the tertiary follicles and the zona parenchymatosa without functional structures showed no correlations between the homologous values from the two measurement areas.

### 3.4. The Size of Mitochondria Decreased with Increasing Age 

Representative images of mitochondria of the two breeds are demonstrated in Figure 1. The results referring to the mitochondrial size (area) for the individual cows are shown in Table 2 and Figure 2. The size of mitochondria decreased with increasing age. However, the influence of age on the size of the mitochondria was not significant (diameter: *p* = 0.509; area: *p* = 0.181). The influence of breed on the size of the mitochondria showed no significant difference from the zero model (diameter: *p* = 0.145; area: *p* = 0.519), with PR having the higher values.

### 3.5. The Length of Telomeres Decreased with Advancing Age

**Figure 3:**
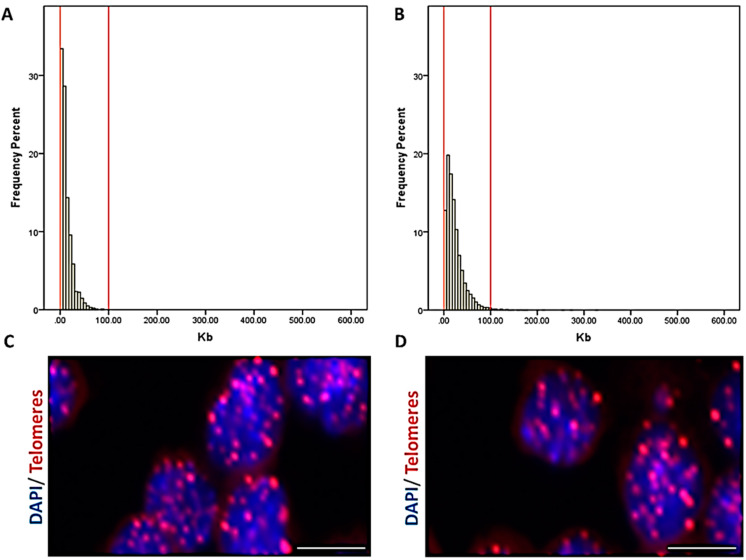
Telomere length in bovine peripheral blood mononuclear cells. **A.** Representative frequency graph of telomere length distribution (a.u.) measured in the Holstein-Friesian cow blood-donor group; the mean telomere length in kb is shown. The red lines are arbitrary lines placed in the exact same position in each frequency graph to visualize differences between samples. **B.** Representative frequency graph of telomere length distribution (a.u.) measured in the Polish Red cow blood donor group; the mean telomere length in kb is shown. The red lines are arbitrary lines placed in the exact same position in each frequency graph to visualize differences between samples. **C.** Representative confocal microscopy picture of visualized telomeres in bovine peripheral blood mononuclear cells of the Holstein-Friesian group (blue = DAPI, red = telomeres, scale bar = 5 µm). **D**. Representative confocal microscopy picture of visualized telomeres in bovine peripheral blood mononuclear cells of the Polish Red group (blue = DAPI, red = telomeres, scale bar = 5 µm).

To address whether telomere shortening occurs at a higher percentage in HF cattle, the breed with high milk production, compared to PR cattle, the dual-purpose (milk/meat) breed with lower milk production, we measured telomere length in peripheral blood mononuclear cells (PBMCs) from all animals. We observed a significant (*p* < 0.001) decrease in the mean telomere length with advancing age (Table 2). The mean percentage of short telomeres, determined in both breeds as telomeres that were in the 10% percentile of the telomere length distribution, was higher in the PBMCs of HF cows when compared with their PR counterparts, although the difference was not significant (*p* = 0.345; R^2^_adj._ 0.931) (Table 1). Frequency graphs of telomere length distribution to visualize the mean telomere length and the number of telomeres that have been analyzed (Figure 3) underline the before-mentioned differences between the cattle breed groups.

## 4. Discussion

The present study is based on the hypothesis that reduced physical resilience in dairy cows of the HPC type is related to activated angiogenesis in reproductive tissues, such as the ovaries. Our assumption was that increased angiogenesis leads to the early exhaustion of the regenerative capacity of HPC and thus, to premature senescence and poorer (re-)productive performance after a short life span characterized by constant, maximal performance. 

Anatomically and functionally, bovine ovaries consist of the zona parenchymatosa (cortex ovarii, ovarian cortex), in which the functional bodies (e.g., follicles and corpora lutea) develop, and a zona vasculosa (medulla ovarii, ovarian medulla), which contains large arteries, veins, lymphatics and nerves. The vascular network acts as a critical physiological regulator by transporting nutrients and oxygen [20]. In addition, blood vessels deliver angiocrine signals that control organ development and stem cell behavior. Tissue-specific capillary beds support the function of each organ and respond to dynamically changing local needs. This is especially true of the endocrine system, which the ovary is a part of [21]. Endocrine glands are highly vascularized, and the vascular system must function optimally to control the rapid and dynamic hormone output [22]. We measured the vascularization of the ovary in the zona parenchymatosa in the two regions (theca folliculi of tertiary follicles and zona parenchymatosa without functional structures) to find out which region would be best suited for measurements. As could be expected, the vascularization of tertiary follicles increased with the increasing size of the follicle, which is a physiological process [23]. The dominant follicle is vascularized best and as such, dependent on successful blood vessel recruitment [24]. Unfortunately, the tertiary follicles were quite scarce in the samples and of varying size. The results of these measurements were thus heterogeneous, which was reflected in the statistical analysis according to age and breed, which had low adjusted R^2^ values and was therefore not meaningful. From this, it is concluded that follicles of comparable size are needed in sufficient quantity for a reliable analysis. The lack of a sufficient number of tertiary follicles for measurement is probably also the explanation for the lack of correlation between the homologous measurements in theca folliculi and zona parenchymatosa without functional structures. The influence of age on the area occupied by capillaries in the zona parenchymatosa without functional structures was significant, but the course of the values (first decrease, then increase) did not appear plausible. The values may have been biased by breed or sample distribution. However, since the distance between the capillaries was also influenced by age and the values were stable here, it can be assumed that age still has an influence on the vascularization of the bovine ovary. Breed influence is also likely, as the Polish Red cow had larger capillaries. From reports in humans, it is known that blood vessel density in the deep cortical stroma significantly decreases in aged women [25]. A decreased blood vessel density was also found in the course of aging in the endocrine system of the mouse, but not in all organs. Interestingly, no change was found in the ovary [26]. An important observation was made by Delgado-Rosas et al. [27], who reported an age-related increase in the superficial cortical stroma vascularization in normal cycling ovaries of woman that was inversely correlated with the density of primordial and primary follicles. Additionally, ovaries from woman with polycystic ovary syndrome showed a 2-fold increase in blood vessel density in both superficial cortical stroma and deep cortical stroma with respect to age-matched controls. The increased vascularization of the superficial cortical stroma in normal older ovaries and in ovaries due to polycystic syndrome could affect the cortical metabolic rate and thus, the survival of the primordial follicles and lead to early follicular growth [25]. Since ovarian cysts in high-performing dairy cows, like the Holstein-Friesian, are also a major factor affecting fertility [28,29,30], these dynamics should be studied more closely. Particular attention should be paid to the spatial distribution of the blood vessels (superficial and deep cortical stroma). To better understand the spatial distribution of vessels and their connection with functional structures in the cow′s ovary, a promising approach could be the imaging of whole organs or bigger blocks of cleared tissues, which makes intact tissue transparent and enables the generation of detailed 3D structures of organs [31,32]. This approach would also allow a detailed analysis of angiogenic and angioregressive figures (e.g., capillary sprouting, dilation, intussusception, thinning, and resorption) that previously required scanning electron microscopy and the preparation of corrosion specimens [33]. In combination with transmission electron microscopy for ultrastructural analysis of endothelial cells, this would enable a comprehensive characterization of the angiogenic processes in the ovary. In addition, differences in vascularization could be associated with differences in follicle development and their ultrastructure. For example, one could look for signs of follicular atresia.

Mitochondrial dysfunction seems to be central to the aging process. As reported by Sharma et al., with increasing age of cells, mitochondrial dysfunction becomes apparent, e.g., manifesting in increased ROS production and the loss of membrane potential. Many of these mitochondrial processes are reflected by their shape [30]. In this study, it was shown that the approximate size of mitochondria decreased with increasing age, but no significant differences have been found so far between the two different breeds, although the trend has been that Polish Red cows have larger mitochondria. We use the term “approximate” size, because the mitochondria were measured, assuming that they have the shape of an ellipse. Of course, this doesn′t really reflect their whole structure, which is supposedly more complex. In age-related pathologies, mitochondria with energy problems have different amounts of fusion and fission proteins. There is a direct relationship between mitochondrial dynamics (ratio of fusion and fission) and mitochondrial function (e.g., energy metabolism and the appropriate distribution of newly synthesized mitochondria during cell division) [34]. However, it is precisely this dynamic nature of the mitochondria, which have to constantly adapt to the changing needs of the cells through fusion and fission, which is difficult to visualize [34]. Since advances have been made in imaging techniques for living cells and tissues, such as the fluorescent labeling of proteins and the three-dimensional reconstruction of mitochondrial electron microscopic images in tissues, these structures became clearer [34]. The use of additional methods could prove to be an asset for further studies of mitochondria in this project. In addition to the structure, the distribution of mitochondria should also be examined.

The aging of cells and organisms is a complex process. Endothelial dysfunction and angiogenesis impairment are key mechanisms by which aging promotes vascular pathologies [35]. It is also assumed that telomere shortening has a negative effect on the aging process due to a restriction of stem cell functionality and regenerative organ reserves [36]. A preterm depletion of the resources for vascular development could have a detrimental effect for ovarian function. In past years, the measurement of telomere length has been commonly performed in PBMCs, since the telomere length in these cells is highly correlated to cells from other tissues of a mammalian organism [37]. Our results concerning the telomere length in PBMCs of both breeds showed a significant shortening of telomeres and a higher percentage of short telomeres with increasing age. This is in line with previous studies [38]. Interestingly, it was observed that the proportion of short telomeres was higher in the PBMCs of HF than in PR, but this was without significance. As an outlook for future studies, it would be interesting to connect the blood levels of anti-Müllerian hormone (AMH) and the antral follicle count (AFC) [39] of different breed dairy cows with the length of telomeres and aging, to decipher the correlation between the before-mentioned factors and to test the hypothesis that AMH is an adequate biomarker for reproductive aging and fertility lifespan.

## 5. Conclusions

We had expected that vascularization might be constantly higher in high-performing cows and then be reduced all at once, i.e., earlier than would be the case in the normal aging process, which would then lead to reproductive failure. However, this line of thought could not be proven in this study, because we could not prove a significant difference of aging parameters between the PR and HF breeds. However, our findings show a tendency for age and breed to influence the capillary blood supply of the bovine ovary, and that the stressor of milk production might enhance premature aging with regards to vascularization. A limitation of the study, so far, was the small number of involved animals. Further studies with increased numbers of animals could show whether the data trends are true and whether there are more differences between the breeds and within breeds correlated to age.

## Figures and Tables

**Table 1:** Vascularization of the tertiary follicles in bovine ovarian tissue. HF Holstein-Friesian, PR Polish Red cow.

Breed	Age in Months	Capillaries per mm^2^	Intercapillary Distance in µm	Capillary Diameter in µm	Capillary Lumen in µm^2^	Area Occupied by Capillaries in %	Area of the Theca Interna Folliculi in mm^2^
HF	62	812	18.32	6.25	36.81	2.99	0.29
HF	73	1361	12.93	6.97	49.98	6.80	0.11
HF	85	1115	12.29	10.61	166.87	18.61	1.68
HF	88	-	-	-	-	-	-
HF	116	861	14.60	9.91	134.81	11.61	0.85
PR	39	1030	16.07	7.67	74.69	6.10	0.24
PR	43	817	15.71	9.51	166.70	13.62	0.32
PR	61	1174	12.40	8.95	91.60	10.75	0.34
PR	90	1200	11.45	9.58	104.29	12.51	0.40
PR	108	1662	10.99	8.29	74.96	12.45	0.10

**Table 2:** Capillary and mitochondria properties in bovine ovarian tissue samples and telomere length and percentile of short telomeres in bovine peripheral blood mononuclear cells. HF Holstein-Friesian, PR Polish Red cow.

Breed	Age in Months	Capillaries per mm^2^	Inter-Capillary Distance in µm	Capillary Diameter in µm	Capillary Lumen in µm^2^	Area Occupied by Capillaries in %	Mito-Chondrial Size (Area in µm^2^)	Telomere Length in kb	Percentile of Short Telomeres
HF	62	903	19.57	8.22	66.36	5.99	0.26	29.75	0.125
HF	73	905	17.64	8.23	67.33	6.09	0.24	23.33	0.147
HF	85	588	17.12	9.70	101.24	5.96	0.19	23.65	0.158
HF	88	614	18.59	9.33	102.19	6.27	0.18	22.78	0.147
HF	116	884	14.40	10.90	141.67	12.52	0.15	12.48	0.692
PR	39	711	16.55	12.17	147.34	10.48	0.26	34.46	0.073
PR	43	604	22.40	9.87	103.19	6.23	0.18	34.78	0.082
PR	61	679	19.37	10.31	114.25	7.76	0.31	27.30	0.088
PR	90	695	17.76	11.04	141.84	9.86	0.23	16.53	0.213
PR	108	687	15.95	10.82	133.30	9.16	0.21	16.54	0.373

## Data Availability

Data are available upon request.
